# A new species of *Micryletta* frog (Microhylidae) from Northeast India

**DOI:** 10.7717/peerj.7012

**Published:** 2019-06-11

**Authors:** Abhijit Das, Sonali Garg, Amir Hamidy, Eric N. Smith, S. D. Biju

**Affiliations:** 1 Wildlife Institute of India, Chandrabani, Dehradun, Uttarakhand, India; 2 Systematics Lab, Department of Environmental Studies, University of Delhi, Delhi, India; 3 Museum Zoologicum Bogoriense, Research Center for Biology, Indonesian Institute of Sciences, Cibinong, West Java, Indonesia; 4 Amphibian and Reptile Diversity Research Center and Department of Biology, University of Texas at Arlington, Arlington, TX, USA

**Keywords:** Amphibia, Phylogeny, South and Southeast Asia, Microhylinae, Systematics, Morphology, Indo-Burma biodiversity hotspot, Taxonomy, Lectotype, Mitochondrial DNA

## Abstract

We describe a new species of frog in the microhylid genus *Micryletta* Dubois, 1987 from Northeast India based on molecular and morphological evidence. The new species, formally described as *Micryletta aishani* sp. nov., is phenotypically distinct from other congeners by a suite of morphological characters such as brown to reddish-brown dorsum; dorsal skin shagreened with minute spinules; snout shape nearly truncate in dorsal and ventral view; a prominent dark streak extending from tip of the snout up to the lower abdomen; ash-grey mottling along the margins of upper and lower lip extending up to the flanks, limb margins and dorsal surfaces of hand and foot; tibiotarsal articulation reaching up to the level of armpits; absence of outer metatarsal tubercles; and absence of webbing between toes. Phylogenetic relationships within the genus are inferred based on mitochondrial data and the new taxon is found to differ from all the recognised *Micryletta* species by 3.5–5.9% divergence in the mitochondrial 16S rRNA. The new species was found in the states of Assam, Manipur, and Tripura, from low to moderate elevation (30–800 m asl) regions lying south of River Brahmaputra and encompassing the Indo-Burma Biodiversity Hotspot. The discovery validates the presence of genus *Micryletta* in Northeast India based on genetic evidence, consequently confirming the extension of its geographical range, westwards from Southeast Asia up to Northeast India. Further, for nomenclatural stability of two previously known species, *Microhyla inornata* (= *Micryletta inornata*) and *Microhyla steinegeri* (= *Micryletta steinegeri*), lectotypes are designated along with detailed descriptions.

## Introduction

The Northeast region of India encompasses two globally recognised biodiversity hotspots—Himalayas in the north and Indo-Burma towards the South (Mittermeier et al., 2004), and is home to unique and diverse array of amphibians (e.g. Pillai & Yazdani, 1973; Kiyasetuo & Khare, 1986; Kamei et al., 2012; Biju et al., 2016). Yet, in comparison to the Western Ghats hotspot in Peninsular India that has witnessed a systematic documentation of amphibian diversity, particularly with over two-fold increase in the number of known species during the past two decades (Biju et al., 2014a; Garg et al., 2017), the Northeast regions of the country have remained relatively neglected (Kamei et al., 2012; Biju et al., 2016). The known amphibian fauna of Northeast India belongs to 11 families (Frost, 2018; AmphibiaWeb, 2018), of which Microhylidae is represented by three genera (*Microhyla* Tschudi, *Kaloula* Gray, and *Uperodon* Duméril and Bibron) confirmed to be present on the basis of detailed morphological and/or molecular studies (e.g. Das et al., 2005; Sengupta et al., 2009; Garg et al., 2018a, 2018b, 2018c). Another enigmatic group of microhylid frogs, genus *Micryletta* Dubois is believed to occur in the northeast state of Manipur based on a cursory report of *Micryletta inornata* Boulenger (Mathew & Sen, 2010). This species was previously also reported from the Andaman Islands of India based on a subadult specimen (Pillai, 1977) that requires confirmation (Harikrishnan & Vasudevan, 2018). Hence, there are uncertainties about the identity of *Micryletta* species in India, and consequently the geographical distribution of the genus and its members (chiefly *M. inornata*) outside of East (Mainland China and Taiwan) and Southeast Asia (Cambodia, Indonesia, Laos, Malaysia, Myanmar, Thailand, and Vietnam) (Poyarkov et al., 2018; Garg & Biju, 2019).

Originally described by Dubois (1987) to accommodate two previously known species, *Microhyla inornata* Boulenger, 1890 (= *Micryletta inornata*) and *Microhyla steinegeri* Boulenger, 1909 (= *Micryletta steinegeri*), genus *Micryletta* remained a taxonomic puzzle for long since its description (Poyarkov et al., 2018); initially the proposal received mixed acceptance with some morphological studies not recognizing the genus (Zhao & Adler, 1993; Fei, 1999) while others accepting it as valid (Orlov et al., 2002; Poyarkov et al., 2014). Eventually, *Micryletta* was shown to be phylogenetically distinct from *Microhyla* (Frost et al., 2006). Although most recent works have shown the placement of the genus in the subfamily Microhylinae (e.g. Van der Meijden et al., 2007; Kurabayashi et al., 2011; Pyron & Wiens, 2011; De Sá et al., 2012; Blackburn et al., 2013; Peloso et al., 2016; Tu et al., 2018; Poyarkov et al., 2018; Garg & Biju, 2019), excepting few mitochondrial datasets (Matsui et al., 2011), its phylogenetic position within the subfamily remained largely debatable until recently (Poyarkov et al., 2018; Garg & Biju, 2019).

Currently, the genus *Micryletta* comprises of four recognised species: (1) *Micryletta inornata*, the type species of the genus (Dubois, 1987), originally described from “Deli, Sumatra” (Boulenger, 1890), subsequently reported widely from regions across mainland Southeast Asia and Indo-Burma, however recently restricted only to the island of Sumatra in Indonesia (Alhadi et al., 2019); (2) *Micryletta steinegeri*, originally described from “Kanshirei” on the island of Taiwan (Boulenger, 1909), tentatively synonymised with *M. inornata* (Parker, 1928) and treated as such (Parker, 1934; Matsui & Busack, 1985), until subsequently removed from synonymy (Dubois, 1987). However, Wang, Wu & Yu (1989) treated Taiwanese populations as *Microhyla inornata* (= *Micryletta inornata*) and recent phylogenetic studies have shown *M. steinegeri* to be close to certain *M*. ‘*inornata*’ populations with shallow divergence (e.g. Matsui et al., 2011; Poyarkov et al., 2018; Garg & Biju, 2019; Alhadi et al., 2019); (3) *Micryletta erythropoda*, originally described from southern Vietnam in the genus *Microhyla* (Tarkhnishvili, 1994), more recently transferred to *Micryletta* (Orlov et al., 2002; Poyarkov et al., 2014), and subsequently reported from adjacent Cambodia (Vassilieva et al., 2016); and (4) *Micryletta nigromaculata*, recently described from two localities in northern Vietnam (Poyarkov et al., 2018). Another taxon was described as a subspecies *Microhyla inornata lineata* from southern Thailand (Taylor, 1962). Although this taxon is currently placed under the synonymy of *Micryletta inornata* (Dubois, 1987), recent studies have suggested that it could represent a distinct species, pending resolution based on molecular data from topotypic specimens (Matsui et al., 2011; Poyarkov et al., 2018; Alhadi et al., 2019).

During surveys over a period of 10 years in Northeast India, our rare encounters with a seemingly secretive species of microhylid frog led us to investigate its identity using molecular and morphological tools. Based on both phenotypic and genotypic evidence, we conclude that this frog belongs to genus *Micryletta* and represents a distinct new species to which no previously available name could be applied. Here, we formally describe the new species based on collections from the states of Assam, Manipur, and Tripura.

## Materials and Methods

### Field surveys and specimen collection

**Figure 1:**
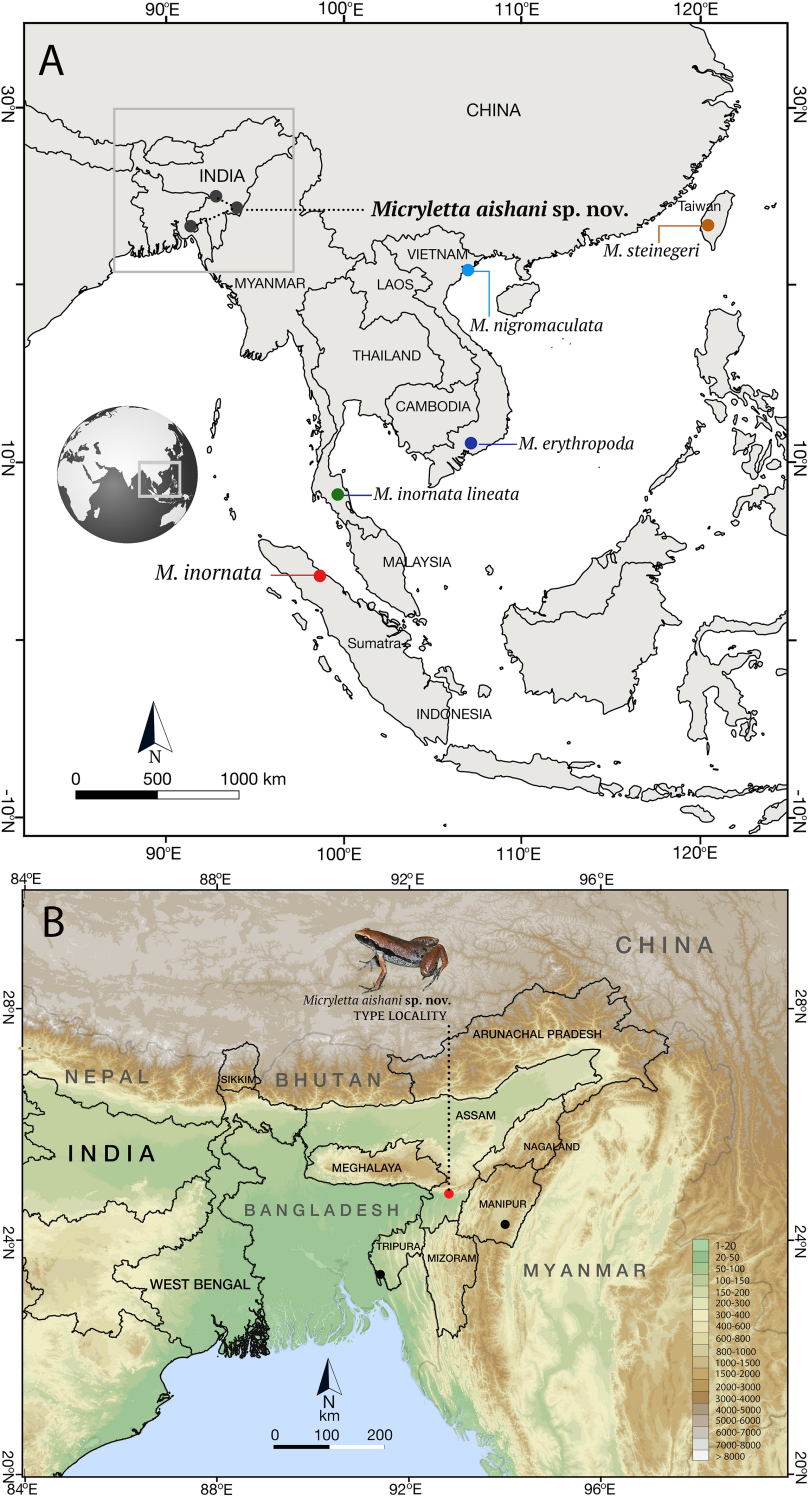
Type localities of species in the genus *Micryletta*. (A) Type localities of previously known taxa from Southeast and East Asia, and the new species from Northeast India. (B) Known distribution of *Micryletta aishani* sp. nov. in Northeast India.

Sampling was carried out at three localities in Northeast India (Fig. 1). Animals were located during opportunistic night searches around water bodies (streams, marshes, stagnant pools, and temporary puddles) inside secondary forests or degraded areas surrounding human settlements. Sampled individuals were photographed, euthanised in MS-222 (Tricaine methane sulphonate), fixed in 4% formalin, and preserved in 70% ethanol. Tissue samples for molecular studies were obtained from the thigh muscle or liver and stored in absolute ethanol at −20 °C. Type specimens are deposited at Zoological Survey of India, Kolkata (ZSIC) and referred specimens are available at Wildlife Institute of India, Dehradun (WII) or Systematics Lab, University of Delhi (SDBDU). Geographical coordinates of the sampling localities were recorded using a Garmin GPS (WGS 84) and distribution maps were prepared in QGIS version 2.6.1 (http://www.qgis.org).

Fieldwork and sampling were carried out with permissions from responsible authorities in the State Forest Departments of Assam, Tripura, and Manipur (Study permits: No. FWLG/455; No. F.8(163)/FOR-WL; No. 3/22/2016-WL). Research received ethical approval from Wildlife Institute of India, Dehradun (WII/ESM/AD/AES-05) and Department of Environmental Studies, University of Delhi (DES/1020), India.

### Morphological study

The new collections were morphologically compared with all previously known *Micryletta* species, based on the original descriptions (Boulenger, 1890, 1909; Taylor, 1962; Tarkhnishvili, 1994; Poyarkov et al., 2018), examination of types and other museum specimens (for *M. inornata* and *M. steinegeri*), and/or type photographs (*M. erythropoda* available from Moscow State University, National Depository Bank of Live Systems website https://depo.msu.ru/, accessed 20 May 2018; *M. inornata lineata* available from FMNH; *M. nigromaculata* available from Poyarkov et al., 2018).

Only adult animals were used for morphometric studies. Sex and maturity of the specimens were determined either by the presence of secondary sexual characters (such as vocal sacs in males; eggs in gravid females) or through gonadal examination with the help of a small lateral or ventral incision. Measurements and photographs were taken for the right side of the specimen, except when a character was damaged, in which case the measurement was taken on the left side. The following measurements were taken to the nearest 0.1 mm using digital slide-calipers or binocular microscope with micrometre ocular: snout-vent length (SVL), head width (HW, at the angle of the jaws), head length (HL, from the rear of the mandible to the tip of the snout), snout length (SL, from the tip of the snout to the anterior orbital border), eye length (EL, horizontal distance between the bony orbital borders), TYD (maximum tympanum diameter), EN (distance from the front of the eye to the nostril), NS (distance from the nostril to the tip of the snout), internarial distance (IN), inter upper eyelid (IUE width, shortest distance between the upper eyelids), UEW (maximum upper eyelid width), forearm length (FAL, from the flexed elbow to the base of the outer palmar tubercle), hand length (HAL, from the base of the outer palmar tubercle to the tip of the third finger), shank length (SHL), thigh length (TL), foot length (FOL, from the base of the inner metatarsal tubercle to the tip of the fourth toe), TFOL (distance from the heel to the tip of the fourth toe), OMTL (length of outer metatarsal tubercle), IMTL (length of inner metatarsal tubercle), FL (finger length, from tip of the digit to its base where it joins the adjacent digit), FD (disc width of finger), FW (width of finger, measured at the base of the disc), TD (disc width of toe), TW (width of toe, measured at the base of the disc); digit number is represented by roman numerals I–V. Number of additional tubercles on toes are represented by roman numerical followed by S (small) or L (large). Morphometric terminologies follow Garg et al. (2018c); description of foot webbing and the webbing formulae follow Biju et al. (2014b). All measurements provided in the taxonomy section are in millimetres (mm).

### Molecular study

Genomic DNA was extracted from a tissue sample of the new species by using Qiagen DNeasy blood and tissue kit (Qiagen, Valencia, CA, USA). A fragment of ∼540 bp of the mitochondrial 16S rRNA gene (16S) was PCR-amplified using previously published primers 16Sar and 16Sbr (Simon et al., 1994). The purified PCR product was sequenced on both strands using BigDye Terminator v3.1 Cycle Sequencing Kit on ABI 3730 automated DNA sequencer (Applied Biosystems, Foster City, CA, USA). Sequences were assembled and checked in ChromasPro v1.34 (Technelysium Pty Ltd., Tewantin, Australia). Additionally, the mitochondrial 16S sequences of all previously published *Micryletta* populations, along with outgroup taxa (Peloso et al., 2016; Garg & Biju, 2019) from five generic representatives of the subfamily Microhylinae (*Glyphoglossus molossus* Günther, *Kaloula pulchra* Gray, *Microhyla achatina* Tschudi, *Mysticellus franki* Garg and Biju, and *Uperodon systoma* (Schneider)) and a member of the subfamily Dyscophinae (*Dyscophus insularis* Grandidier) were retrieved from the GenBank (Table 1). A dataset of total 44 taxa was assembled, aligned using ClustalW, and manually optimised in MEGA 6.0 (Tamura et al., 2013). The new sequence is deposited in the GenBank under accession number MK889218.

**Table 1:** List of samples used for the molecular study.

	Species	Locality	Voucher/Source	Accession No.	Reference
Ingroup					
1	*Micryletta aishani* sp. nov.	India: Assam, Cachar district, Subhong	SDBDU 3920	MK889218	Present study
2	*Micryletta erythropoda*	Vietnam: Dong Nai, Ma Da (Vinh Cuu) N.R.	ZMMU A4721-1533	MH756146	Poyarkov et al. (2018)
3	*Micryletta erythropoda*	Vietnam: Dong Nai, Ma Da (Vinh Cuu) N.R.	ZMMU A4721-1542	MH756147	Poyarkov et al. (2018)
4	*Micryletta inornata*	Indonesia: Sumatra, North Sumatra, Deli Serdang	MZB Amph 23947	LC208136	Alhadi et al. (2019)
5	*Micryletta inornata*	Indonesia: Sumatra, North Sumatra, Deli Serdang	MZB Amph 23948	LC208137	Alhadi et al. (2019)
6	*Micryletta inornata*	Indonesia: Sumatra, North Sumatra, Deli Serdang	MZB Amph 23949	LC208135	Alhadi et al. (2019)
7	*Micryletta inornata*	Indonesia: Sumatra, Aceh	MZB Amph 27242	LC208138	Alhadi et al. (2019)
8	*Micryletta* cf. *inornata lineata*	Myanmar: Tanintharyi Div., Kawthaung dist.	CAS 247206	KM509167	Peloso et al. (2016)
9	*Micryletta* cf. *inornata lineata*	Thailand: Ranong	KUHE 23858	AB634695	Matsui et al. (2011)
10	*Micryletta nigromaculata*	Vietnam: Hai Phong, Cat Ba N.P.	ZMMU A5947	MH756148	Poyarkov et al. (2018)
11	*Micryletta nigromaculata*	Vietnam: Hai Phong, Cat Ba N.P.	ZMMU A5937	MH756149	Poyarkov et al. (2018)
12	*Micryletta nigromaculata*	Vietnam: Hai Phong, Cat Ba N.P.	ZMMU A5934	MH756150	Poyarkov et al. (2018)
13	*Micryletta nigromaculata*	Vietnam: Hai Phong, Cat Ba N.P.	ZMMU A5940	MH756152	Poyarkov et al. (2018)
14	*Micryletta nigromaculata*	Vietnam: Hai Phong, Cat Ba N.P.	ZMMU A5946	MH756151	Poyarkov et al. (2018)
15	*Micryletta nigromaculata*	Vietnam: Hai Phong, Cat Ba N.P.	ZMMU A5942	MH756153	Poyarkov et al. (2018)
16	*Micryletta nigromaculata*	Vietnam: Ninh Binh, Cuc Phuong N.P.	DTU 301	MH756154	Poyarkov et al. (2018)
17	*Micryletta nigromaculata*	Vietnam: Ninh Binh, Cuc Phuong N.P.	DTU 303	MH756155	Poyarkov et al. (2018)
18	*Micryletta nigromaculata*	Vietnam: Ninh Binh, Cuc Phuong N.P.	DTU 304	MH756156	Poyarkov et al. (2018)
19	*Micryletta* cf. *nigromaculata*	Laos: Thaphabat, Bolikhamxay	FMNH:255123	KC822493	Blackburn et al. (2013)
20	*Micryletta steinegeri*	Taiwan: Yunlin	KUHE 35937	AB634696	Matsui et al. (2011)
21	*Micryletta* cf. *steinegeri*	Vietnam: Ninh Binh, Cuc Phuong N.P.	DTU 310	MH879840	Poyarkov et al. (2018)
22	*Micryletta* cf. *steinegeri*	Vietnam: Ninh Binh, Cuc Phuong N.P.	DTU 311	MH879841	Poyarkov et al. (2018)
23	*Micryletta* cf. *steinegeri*	Vietnam: Ninh Binh, Cuc Phuong N.P.	DTU 312	MH879842	Poyarkov et al. (2018)
24	*Micryletta* cf. *steinegeri*	Vietnam: Hai Phong, Cat Ba N.P.	ZMMU NAP-3352-1	MH879843	Poyarkov et al. (2018)
25	*Micryletta* cf. *steinegeri*	Vietnam: Hai Phong, Cat Ba N.P.	ZMMU NAP-3352-2	MH879844	Poyarkov et al. (2018)
26	*Micryletta* cf. *steinegeri*	Vietnam: Hai Phong, Cat Ba N.P.	ZMMU NAP-3580	MH879845	Poyarkov et al. (2018)
27	*Micryletta* sp. A	Laos: Boulapha, Khammouan	FMNH 255121	KC179997	De Sá et al. (2012)
28	*Micryletta* sp. A	Laos: Boualapha, Khammouan Prov.	FMNH:255121	KC822494	Blackburn et al. (2013)
29	*Micryletta* sp. A	Laos: Ban Sop Chuna, Luangprabang Prov.	K3246	KC180027	De Sá et al. (2012)
30	*Micryletta* sp. A	Laos: Long Nai Kao, Phongsaly Province	K1956	KC180067	De Sá et al. (2012)
31	*Micryletta* sp. A	Laos: Luang Prabang, Ban Sop Choun	2006.2401	KR827951	Grosjean et al. (2015)
32	*Micryletta* sp. A	Laos: Phongsaly, Long Nai Khao	2005.0179	KR827952	Grosjean et al. (2015)
33	*Micryletta* sp. A	Thailand: Chiang Mai, Doi Chiang Dao	K3068	KR827953	Grosjean et al. (2015)
34	*Micryletta* sp. A	Thailand: Phrae, Mae Yom	KUHE 20497	AB598341	Matsui et al. (2011)
35	*Micryletta* sp. A	Thailand: (no locality record)	NA (Note: No. X21655)	AF215375	Vences (2000)
36	*Micryletta* sp. A	Vietnam: Ha Tinh, Ke Go	TZ9892	AF285206	Ziegler (2000)
37	*Micryletta* sp. A	Vietnam: Ha Tinh, Ke Go	TZ98110	AF285207	Ziegler (2000)
38	*Micryletta* sp. B	Laos: (no locality record)	KUHE 35133	AB611968	Kurabayashi et al. (2011)
Outgroup					
39	*Glyphoglossus molossus*	Myanmar: Sagaing	CAS 210056	KM509135	Peloso et al. (2016)
40	*Kaloula pulchra*	China	NMNS 3208	KC822614	Blackburn et al. (2013)
41	*Microhyla achatina*	Indonesia: Java, Ungaran	MZB Amp 16402	KM509162	Peloso et al. (2016)
42	*Mysticellus franki*	India: Kerala, Wayand	ZSI/WGRC/V/A/967	MK285340	Garg & Biju (2019)
43	*Uperodon systoma*	India: Tamil Nadu: Kunnapattu	SDBDU 2005.4723	MG557949	Garg et al. (2018b)
44	*Dyscophus insularis*	Madagascar	AMNH-A 173883	KM509128	Peloso et al. (2016)

Maximum likelihood (ML) and Bayesian analyses were performed to reconstruct phylogenetic relationships. The appropriate model of sequence evolution was determined by implementing Akaike Information Criteria in ModelTest 3.4 (Posada & Crandall, 1998). ML searches were executed in PAUP* (Swofford, 2002) using the best-fit model (GTR+I+G) with all parameters estimated. Bayesian analysis was implemented in MrBayes (Ronquist & Huelsenbeck, 2003) using uniform priors and four Metropolis-Coupled Markov Chain Monte Carlo chains for 25 million generations. Trees were sampled after every 1,000 generations. Convergence of the runs was determined by average standard deviation of the split frequencies of <0.01 and potential scale reduction factors of ∼1.0, and stationarity was observed through log likelihood trends. Bayesian posterior probabilities (BPP) for the clades were summarised after discarding the first 20% trees as burn-in (Huelsenbeck et al., 2001). Clade support was also assessed by 10,000 rapid bootstrap replicates executed using GTRGAMMA model in RAxML 7.3.0 (Stamatakis, Hoover & Rougemont, 2008) as implemented in raxmlGUI 1.1 (Silvestro & Michalak, 2012). Uncorrected pairwise distances were computed in PAUP* (Swofford, 2002) to understand intra and interspecific genetic divergences. A Median-Joining network was constructed using the software Network 4.6.1.0 (www.fluxus-engineering.com). A 178 bp fragment available for all the 16S *Micryletta* sequences was used to evaluate relationships and possible mutation steps among 21 haplotypes recovered from 38 samples.

### Nomenclatural acts

The electronic version of this article in portable document format will represent a published work according to the International Commission on Zoological Nomenclature (ICZN), and hence the new names contained in the electronic version are effectively published under that Code from the electronic edition alone (see Articles 8.5–8.6 of the Code). This published work and the nomenclatural acts it contains have been registered in ZooBank, the online registration system for the ICZN. The ZooBank Life Science Identifiers (LSIDs) can be resolved and the associated information can be viewed through any standard web browser by appending the LSID to the prefix http://zoobank.org/. The LSID for this publication is as follows: urn:lsid:zoobank.org:pub:F0C54D63-0B29-470F-BDCD-72F7E2511C51. The online version of this work is archived and available from the following digital repositories: PeerJ, PubMed Central, and CLOCKSS.

## Results

### Genetic relationships and sequence divergence

**Figure 2:**
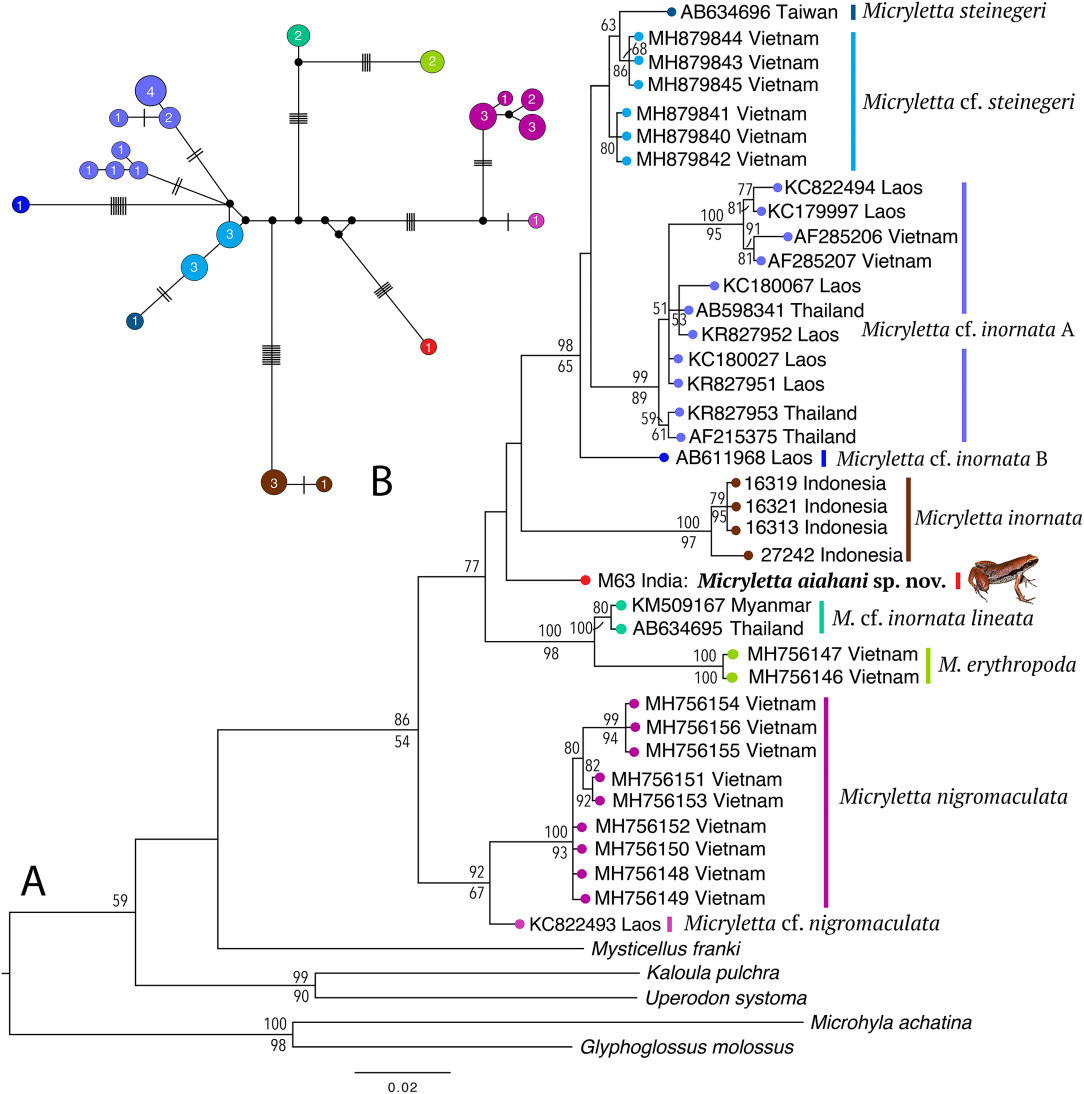
Molecular relationships in the genus *Micryletta* based on mitochondrial 16S rRNA sequences. (A) Maximum Likelihood tree showing the phylogenetic position of *Micryletta aishani* sp. nov. and relationships among all the known and candidate species in the genus. Bayesian Posterior Probabilities and RAxML bootstrap values >50% are indicated above and below the branches, respectively. Accession numbers are cross-referenced in Table 1. (B) Median-Joining network reconstructed from 21 haplotypes representing 38 *Micryletta* samples. Circle colours correspond with clade labels indicated for each taxon on the ML tree. Circle sizes are proportional to the number of haplotype sequences involved, as indicated by the circle numbers. Black circles represent median vectors. Each branch represents one mutation step; black bars represent additional mutation steps.

Phylogenetically, the new frog is nested in the genus *Micryletta* where it forms a clearly divergent lineage (Fig. 2) with >3% divergence from all previously known species in the studied fragment of the mitochondrial 16S rRNA (Table 2). The analyses recovered 10 distinct sub-clades within the genus, representing four recognised species (*M. inornata*, *M. erythropoda*, *M. steinegeri*, and *M. nigromaculata*), a possibly previously available name (*M*. cf. *inornata lineata*), the new *Micryletta* population from Northeast India (formally described as *M. aishani* sp. nov.), and four other potential candidate species (*Micryletta* sp. A, *Micryletta* sp. B, *M*. cf. *steinegeri*, and *M*. cf. *nigromaculata*). The *Micryletta* sample from Northeast India was more closely related to *M. inornata*, *M. steinegeri* species complex (*M. steinegeri*, *M*. cf. *steinegeri*, *Micryletta* sp. A, and *Micryletta* sp. B), and the clade comprising of *M. erythropoda* + *M*. cf. *inornata lineata*, than to *M. nigromaculata* + *M*. cf. *nigromaculata*. The distinct phylogenetic position of *M. aishani* sp. nov. was concordantly recovered in all our analyses, however the relationships among the four closely related and otherwise well-supported major lineages—*M. aishani* sp. nov., *M. inornata* (BPP 100, BS 97), *M. steinegeri* species complex (BPP 98, BS 65), and *M. erythropoda* + *M*. cf. *inornata lineata* (BPP 100, BS 98), were poorly resolved. However, our overall topology was largely consistent with the previous studies that include most of the available *Micryletta* sequence data (Poyarkov et al., 2018; Alhadi et al., 2019).

**Table 2:** Inter and intraspecific uncorrected *p*-distances (in percent) for the mitochondrial 16S rRNA gene sequences.

Species	*Micryletta aishani* sp. nov.	*Micryletta erythropoda*	*Micryletta inornata*	*Micryletta* cf. *inornata lineata*	*Micryletta nigromaculata*	*Micryletta* cf. *nigromaculata*	*Micryletta steinegeri*	*Micryletta* cf. *steinegeri*	*Micryletta* sp. A	*Micryletta* sp. B
*Micryletta aishani* sp. nov.	–									
*Micryletta erythropoda*	4.7	0								
*Micryletta inornata*	4.9 (4.5–5.9)	7.3 (7.0–8.2)	0.9 (0–1.7)							
*Micryletta* cf. *inornata lineata*	3.3	2.5	6.3 (5.9–7.3)	0						
*Micryletta nigromaculata*	4.8 (4.5–5.1)	7.8 (7.4–8.3)	6.4 (5.4–7.8)	5.8 (5.3–6.5)	0.7 (0–1.4)					
*Micryletta* cf. *nigromaculata*	4.1	7.3	6.9 (6.2–9.1)	5.4	3.2 (2.3–4.4)	–				
*Micryletta steinegeri*	3.5	5.5	5.3 (5.0–6.1)	4.1	5.4 (5.1–5.8)	6.2	–			
*Micryletta* cf. *steinegeri*	3.2 (3.1–3.2)	5.0 (4.9–5.1)	5.2 (5.0–6.1)	3.7 (3.1–4.2)	5.7 (5.1–6.5)	5.8 (5.0–6.6)	1.3 (1.2–1.4)	0.3 (0–0.5)		
*Micryletta* sp. A	4.0 (3.1–4.8)	6.0 (5.3–6.8)	6.9 (5.7–9.4)	4.9 (4.1–5.8)	6.6 (5.5–7.9)	6.0 (5.4–7.4)	2.7 (2.0–3.4)	2.6 (1.9–3.3)	1.4 (0–3.0)	
*Micryletta* sp. B	5.7	5.7	5.2 (4.9–6.1)	4.5	6.0 (5.7–6.3)	6.9	2.7	2.3 (2.3–2.4)	3.4 (2.7–3.9)	–

**Note:**

Values indicate mean genetic distances with minimum and maximum distances in parenthesis.

The Median-Joining network based on 21 unique haplotypes representing 38 *Micryletta* sequences also recovered *M. aishani* sp. nov. as a distinct species (Fig. 2). All the major lineages and species-level clades observed in our phylogenetic analysis were demarcated as distinct clusters with similar haplotype relationships, consequently providing additional evidence for our findings. The haplotype clusters for all the known and potential candidate species were separated by minimum of four up to 18 mutation steps (hereafter steps) from closely related members, except *M. steinegeri* and *M*. cf. *steinegeri* that were separated by three steps. *M. aishani* sp. nov. was most closely linked to the *M. steinegeri* species complex (specifically *M*. cf. *steinegeri*) with minimum 10 steps, followed by minimum 14 steps each with *M. steinegeri*, *M. erythropoda*, and *M*. cf. *nigromaculata*, and minimum 18 steps each with *M. inornata* and *M. nigromaculata*.

The uncorrected sequence divergence in the studied 16S fragment showed *M. aishani* sp. nov. to be divergent from the previously recognised species by interspecific genetic distances of 3.5% for *M. steinegeri*, 4.5–5.9% for *M. inornata*, 4.7% for *M. erythropoda*, and 4.5–5.1% from *M. nigromaculata*. It was also divergent from the populations suspected to represent another available nomen *M*. cf. *inornata lineata* by 3.3%. The new species also showed a minimum of 3.1% up to 4.8% divergence from other potential candidate species identified in the genus (Table 2). The trends of interspecific divergences between other congeners ranged from minimum 2.5% between *M. erythropoda* and *M*. cf. *inornata lineata*, up to a maximum of 9.1% and 9.4% (between *M. inornata*–*M. steinegeri* species complex and *M. inornata*–*M*. cf. *nigromaculata*, respectively). The *M. steinegeri* species complex was, however, an exception as it showed shallow interspecific distances of 1.2–2.7% among four closely related clades (*M. steinegeri*, *M*. cf. *steinegeri*, *Micryletta* sp. A, and *Micryletta* sp. B), as well as high intraspecific distances of up to 3.0% within the *Micryletta* sp. A clade, suggesting that all of these either represent a single widely distributed species or a complex of multiple taxa with at least two potential candidate species. Another candidate species, *M*. cf. *nigromaculata* with 2.3–4.4% divergence from *M. nigromaculata*, was identified. Further phylogenetic studies complemented with proper identification as well as morphological comparison among all the *Micryletta* populations known so far will be necessary to ascertain the taxonomic status of the yet unidentified populations as well as to understand the patterns of genetic differentiation within the genus.

### Description of new species

**Figure 3:**
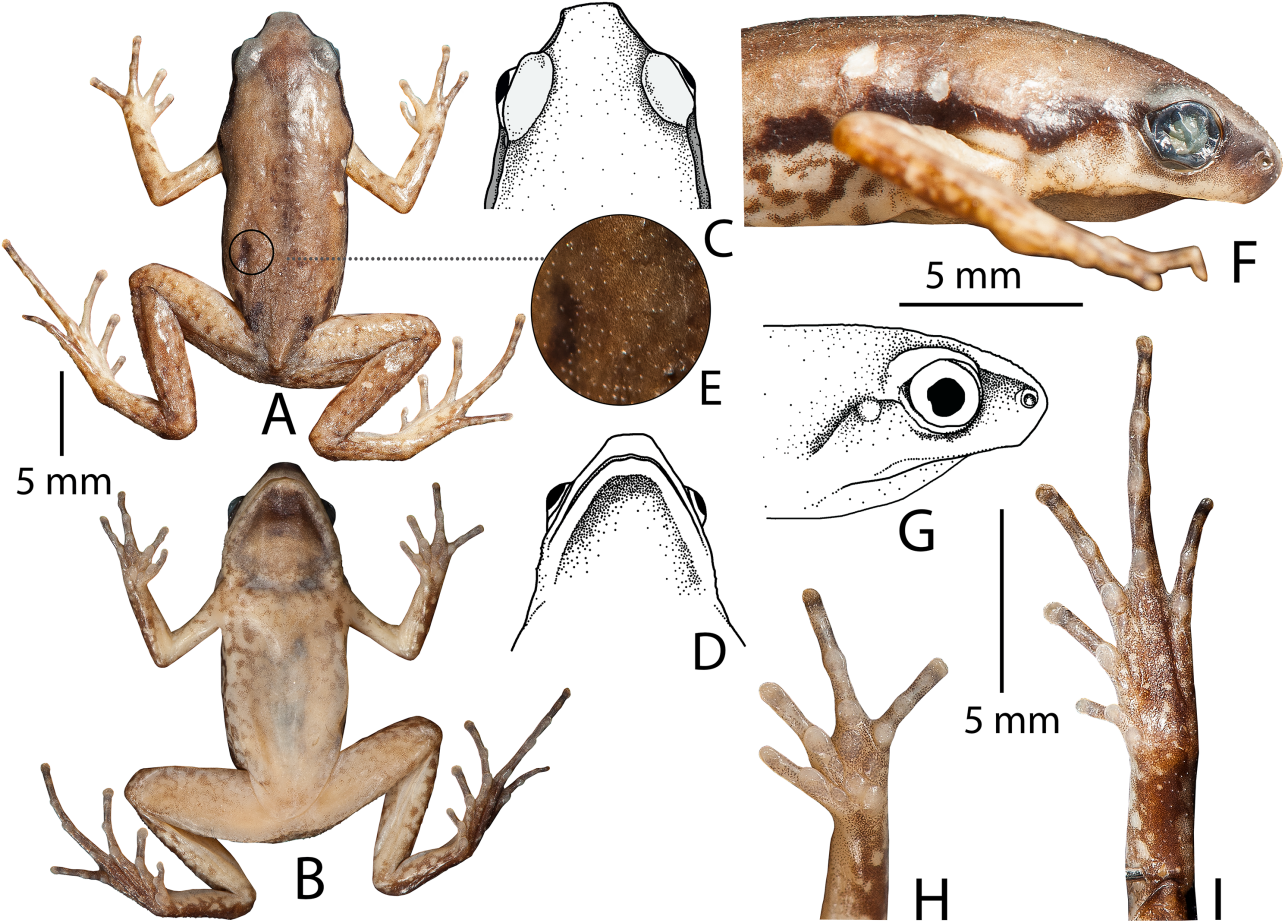
*Micryletta aishani* sp. nov. (ZSIC 14304, HT) in preservation. (A) Dorsal view. (B) Ventral view. (C) Dorsal view of head. (D) Ventral view of head. (E) Magnified view of dorsal skin texture. (F–G) Lateral view of head. (H) Ventral view of hand. (I) Ventral view of foot.

**Figure 4:**
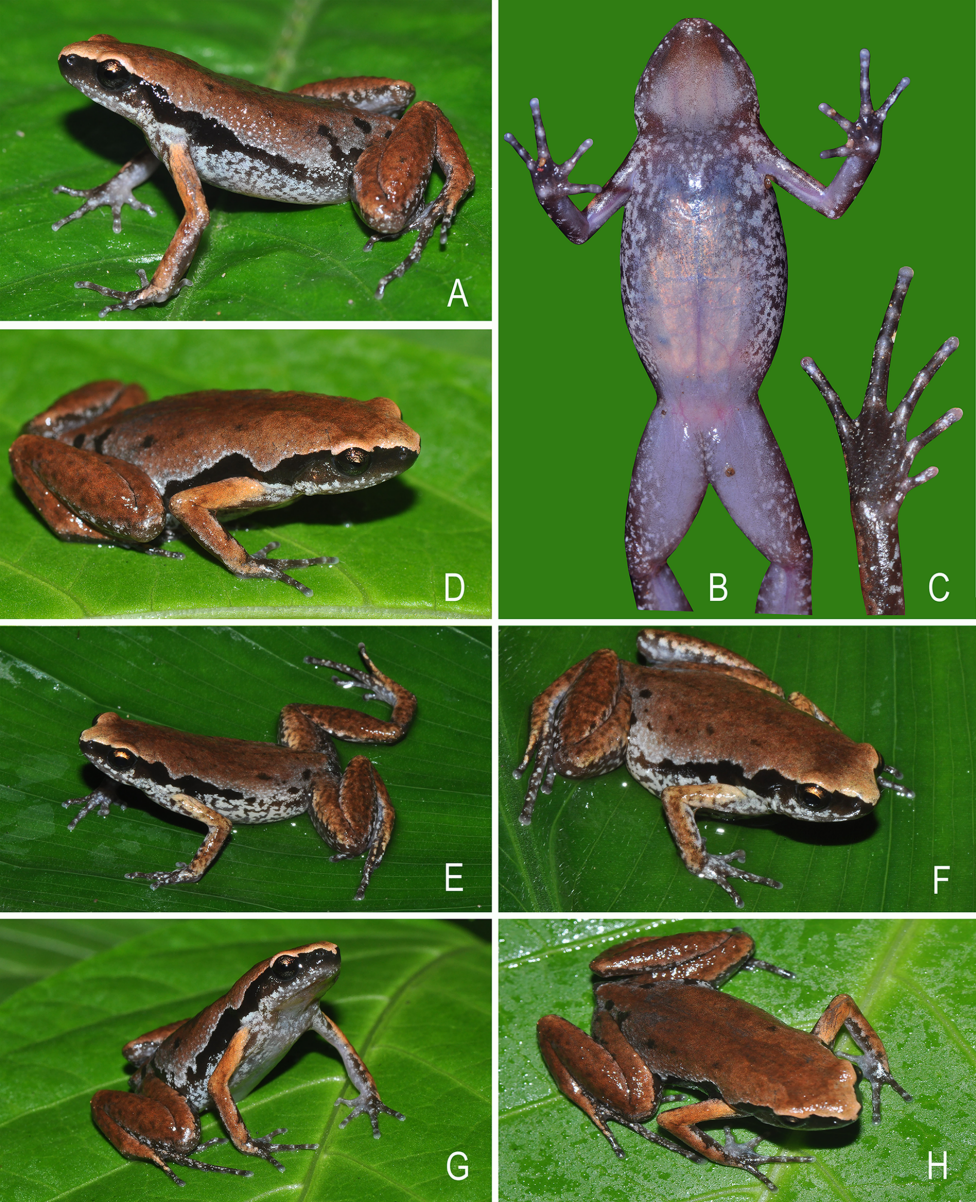
Holotype and paratype specimens of *Micryletta aishani* sp. nov. in life. (A) Dorsolateral view (ZSIC 14304, HT). (B) Ventral view (ZSIC 14304, HT). (C) Ventral view of foot (ZSIC 14304, HT). (D) Dorsolateral view (ZSIC 14305, PT). (E) Dorsolateral view showing lateral and groin markings (ZSIC 14310, PT). (F) Dorsolateral view (ZSIC 14310, PT). (G) Frontolateral view (ZSIC 14311, PT). (H) Dorsal view (ZSIC 14311, PT).

**Figure 5:**
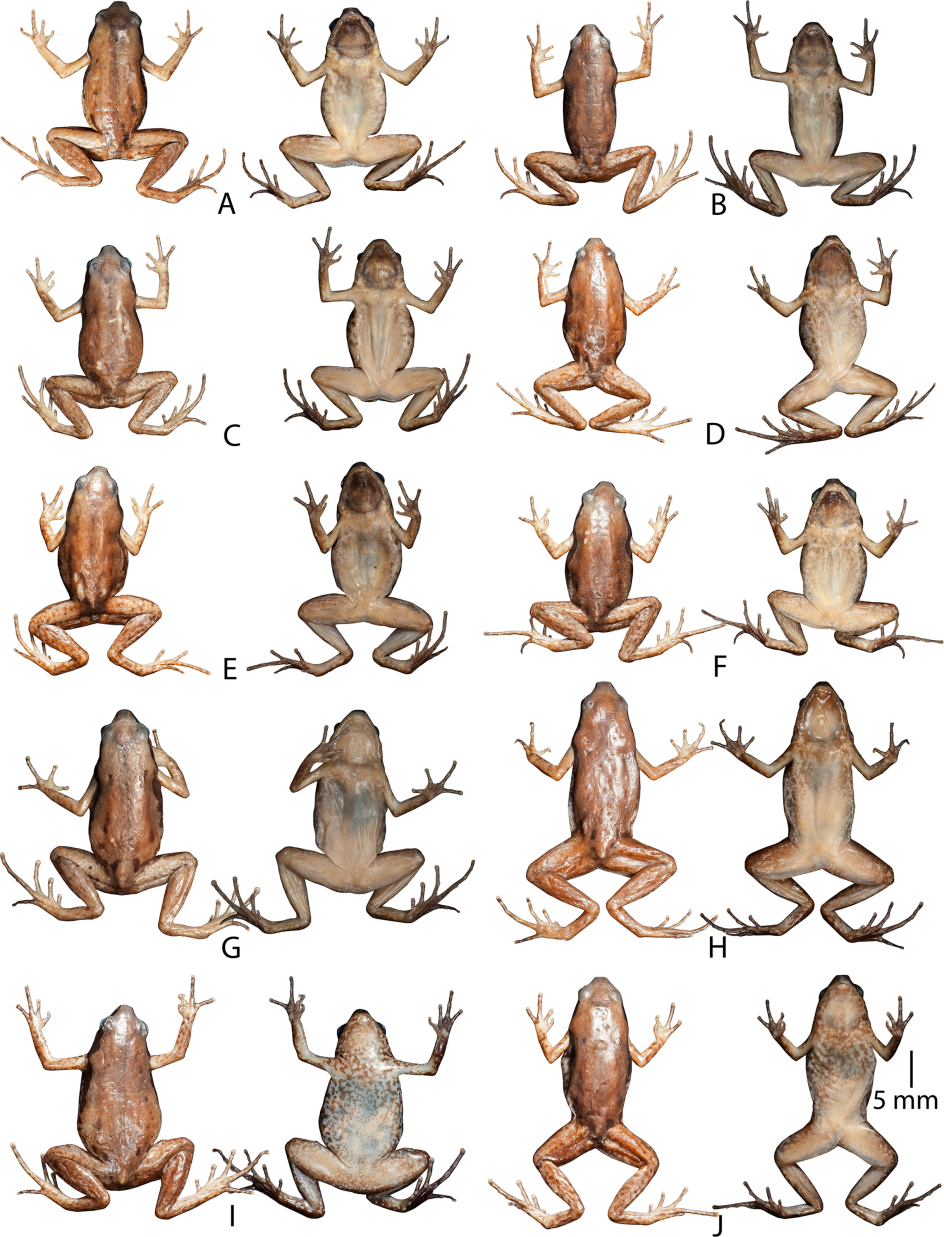
Dorsal and ventral view of paratype and referred specimens of *Micryletta aishani* sp. nov. in preservation. (A) ZSIC 14305 (PT, male). (B) ZSIC 14308 (PT, male). (C) ZSIC 14309 (PT, male). (D) ZSIC 14306 (PT, male). (E) ZSIC 14307 (PT, male). (F) SDBDU 3918 (RS, male). (G) ZSIC 14312 (PT, female). (H) ZSIC 14311 (PT, female). (I) ZSIC 14310 (PT, female). (J) SDBDU 3919 (RS, female).

***Micryletta aishani* sp. nov**.
Zoobank urn:lsid:zoobank.org:act:A8DCDA95-6D6A-4710-9C4D-FC00881032EE
Northeast Indian Paddy Frog
(Figs. 1–5; Tables 1–3)

**Table 3:** Morphometric measurements of *Micryletta* species described in the text (*Micryletta aishani* sp. nov., *Micryletta inornata*, and *Micryletta steinegeri*).

*Micryletta aishani* sp. nov.												
Voucher No	Status	Sex	SVL	HW	HL	SL	EL	TYD	EN	NS	IUE	UEW
ZSIC 14304	HT	M	22.8	6.2	5.7	2.7	2.2	0.4	1.2	0.9	2.7	1.3
ZSIC 14305	PT	M	22.1	6.0	5.5	2.8	2.1	0.3	1.3	1.0	3.3	1.3
ZSIC 14306	PT	M	22.0	6.0	5.8	2.5	2.0	0.4	1.2	0.7	2.8	1.1
ZSIC 14307	PT	M	22.9	6.7	6.5	2.8	2.2	0.5	1.3	0.8	2.9	1.3
ZSIC 14308	PT	M	23.5	6.0	6.0	2.6	2.1	0.6	1.3	0.7	2.6	1.1
ZSIC 14309	PT	M	23.2	6.1	5.6	2.8	2.3	0.4	1.2	0.8	3.0	1.3
SDBDU 3918	RS	M	22.8	6.1	5.8	3.0	2.4	0.5	1.3	1.0	2.6	1.3
		Mean	22.8	6.2	5.8	2.7	2.2	0.4	1.3	0.8	2.8	1.2
		SD	0.5	0.3	0.3	0.2	0.1	0.1	0.1	0.1	0.3	0.1

Voucher No	Status	Sex	IN	FAL	HAL	FIL	FIIL	FIIIL	FIVL	TL	SHL	FOL
ZSIC 14304	HT	M	1.6	5.3	5.9	1.5	2.2	3.6	2.4	10.4	10.7	11.4
ZSIC 14305	PT	M	1.5	5.4	6.0	1.7	2.3	3.6	2.5	10.2	10.3	11.2
ZSIC 14306	PT	M	1.3	5.0	5.6	1.5	1.9	3.5	2.1	9.4	10.1	10.6
ZSIC 14307	PT	M	1.6	5.0	5.7	1.5	2.1	3.8	2.2	9.8	10.3	11.1
ZSIC 14308	PT	M	1.8	5.6	6.0	1.4	1.9	3.6	2.2	10.3	10.5	11.8
ZSIC 14309	PT	M	1.5	5.2	5.5	1.5	1.9	3.2	2.3	10.1	10.5	10.7
SDBDU 3918	RS	M	1.3	5.2	5.8	1.6	2.3	3.4	2.5	10.0	10.4	10.6
		Mean	1.5	5.2	5.8	1.5	2.1	3.5	2.3	10.0	10.4	11.1
		SD	0.2	0.2	0.2	0.1	0.2	0.2	0.2	0.3	0.2	0.5

Voucher No	Status	Sex	SVL	HW	HL	SL	EL	TYD	EN	NS	IUE	UEW
ZSIC 14310	PT	F	27.3	7.3	7.2	2.8	2.6	0.9	1.3	0.9	3.5	1.2
ZSIC 14311	PT	F	26.5	7.4	7.1	3.0	2.2	0.9	1.4	0.9	3.1	1.2
ZSIC 14312	PT	F	26.4	7.1	7.1	2.9	2.5	0.8	1.2	0.8	3.3	1.2
SDBDU 3919	RS	F	25.6	6.8	5.9	2.8	2.1	0.8	1.3	0.8	2.9	1.3
		Mean	26.5	7.2	6.8	2.9	2.4	0.9	1.3	0.9	3.2	1.2
		SD	0.7	0.3	0.6	0.1	0.2	0.1	0.1	0.1	0.3	0.1

Voucher No	Status	Sex	IN	FAL	HAL	FIL	FIIL	FIIIL	FIVL	TL	SHL	FOL
ZSIC 14310	PT	F	1.7	6.5	7.1	1.8	2.3	4.2	2.5	12.3	12.5	13.0
ZSIC 14311	PT	F	1.7	5.8	6.5	1.8	2.1	3.8	2.5	11.4	12.0	12.4
ZSIC 14312	PT	F	1.6	5.9	7.1	1.8	2.3	4.6	3.1	11.5	11.8	12.5
SDBDU 3919	RS	F	1.6	5.7	6.3	1.6	2.4	4.3	2.7	10.3	10.8	11.5
		Mean	1.7	6.0	6.8	1.8	2.3	4.2	2.7	11.4	11.8	12.4
		SD	0.1	0.4	0.4	0.1	0.1	0.3	0.3	0.8	0.7	0.6

*Micryletta inornata*												
Voucher No	Status	Sex	SVL	HW	HL	SL	EL	TYD	EN	NS	IUE	UEW
NHM 1889.11.12.4 (ex. BMNH 1947.2.11.74)	LT	F	19.5	5.9	5.3	2.2	2.4	1.0	1.3	0.8	2.5	1.3

Voucher No	Status	Sex	IN	FAL	HAL	FIL	FIIL	FIIIL	FIVL	T L	SHL	FOL
NHM 1889.11.12.4 (ex. BMNH 1947.2.11.74)	LT	F	1.5	4.8	4.7	1.1	1.5	4.1	2.3	8.1	8.6	8.6

Voucher No	Status	Sex	SVL	HW	HL	SL	EL	TYD	EN	NS	IUE	UEW
NHM 1889.11.12.30 (ex. BMNH 1947.2.11.75)	PLT	M	16.8	4.9	4.6	1.8	2.1	0.6	1.1	0.5	2.1	0.8
MZB Amph 27242	TT	M	20.5	5.7	5.1	2.1	2.3	0.7	1.5	0.7	2.7	1.1
MZB Amph 8542	TT	M	17.0	5.6	4.8	1.9	2.1	0.6	1.0	0.5	2.4	1.0
		Mean	18.1	5.4	4.8	1.9	2.2	0.6	1.2	0.6	2.4	1.0
		SD	1.7	0.4	0.2	0.1	0.1	0	0.2	0.1	0.2	0.1

Voucher No	Status	Sex	IN	FAL	HAL	FIL	FIIL	FIIIL	FIVL	TL	SHL	FOL
NHM 1889.11.12.30 (ex. BMNH 1947.2.11.75)	PLT	M	1.5	3.8	4.1	1.0	1.3	4.2	1.8	6.9	7.8	7.8
MZB Amph 27242	TT	M	1.4	4.4	5.0	1.2	1.5	4.7	2.6	8.5	9.0	8.9
MZB Amph 8542	TT	M	1.3	4.1	4.5	1.1	1.3	4.4	2.0	7.1	8.5	8.7
		Mean	1.4	4.1	4.5	1.1	1.4	4.4	2.1	7.5	8.4	8.5
		SD	0.1	0.2	0.4	0.1	0.1	0.3	0.4	0.9	0.6	0.6

*Micryletta steinegeri*												
Voucher No	Status	Sex	SVL	HW	HL	SL	EL	TYD	EN	NS	IUE	UEW
NHM 1909.10.29.92 (ex. BMNH 1947.2.11.76)	LT	F	30.1	8.4	7.8	2.9	3.3	0.9	1.5	1.0	3.8	1.8
NHM 1909.10.29.93 (ex. BMNH 1947.2.11.77)	PLT	F	27.0	7.7	7.1	2.7	2.9	0.8	1.6	0.9	3.4	1.4
		Mean	28.6	8.1	7.5	2.8	3.1	0.9	1.6	1.0	3.6	1.6
		SD	2.2	0.5	0.5	0.1	0.3	0.1	0.1	0.1	0.3	0.3

Voucher No	Status	Sex	IN	FAL	HAL	FIL	FIIL	FIIIL	FIVL	TL	SHL	FOL
NHM 1909.10.29.92 (ex. BMNH 1947.2.11.76)	LT	F	2.1	6.8	8.2	2.9	3.8	5.4	3.2	12.1	13.0	13.6
NHM 1909.10.29.93 (ex. BMNH 1947.2.11.77)	PLT	F	1.9	6.3	6.8	2.4	3.1	4.8	2.6	10.3	11.7	11.6
		Mean	2.0	6.6	7.5	2.7	3.5	5.1	2.9	11.2	12.4	12.6
		SD	0.1	0.4	1.0	0.4	0.5	0.4	0.4	1.3	0.9	1.4

Voucher No	Status	Sex	SVL	HW	HL	SL	EL	TYD	EN	NS	IUE	UEW
NHM 1909.10.29.94 (ex. BMNH 1947.2.11.78)	PLT	M	22.6	7.6	6.9	2.5	2.6	0.8	1.2	0.7	3.1	1.6
NHM 1909.10.29.95 (ex. BMNH 1947.2.11.79)	PLT	M	23.3	7.5	7.3	2.5	2.6	0.6	1.4	0.6	3.2	1.3
NHM 1909.10.29.96 (ex. BMNH 1947.2.11.80)	PLT	M	23.5	7.3	6.8	2.6	2.7	0.6	1.3	0.8	3.2	1.4
		Mean	23.1	7.5	7.0	2.5	2.6	0.7	1.3	0.7	3.2	1.4
		SD	0.5	0.2	0.3	0.1	0.1	0.1	0.1	0.1	0.1	0.2

Voucher No	Status	Sex	IN	FAL	HAL	FIL	FIIL	FIIIL	FIVL	TL	SHL	FOL
NHM 1909.10.29.94 (ex. BMNH 1947.2.11.78)	PLT	M	1.8	5.7	6.2	1.9	2.6	4.2	2.5	9.3	10.1	11.2
NHM 1909.10.29.95 (ex. BMNH 1947.2.11.79)	PLT	M	1.6	5.6	6.1	1.8	2.3	3.9	2.2	8.8	10.1	11.1
NHM 1909.10.29.96 (ex. BMNH 1947.2.11.80)	PLT	M	1.8	6.6	6.2	1.9	3.1	3.9	2.7	10.3	11.0	11.1
		Mean	1.8	6.0	6.2	1.9	2.7	4.0	2.5	9.5	10.4	11.1
		SD	0.2	0.6	0.1	0.1	0.4	0.2	0.3	0.8	0.5	0.1

**Note:**

Measurement abbreviations and museum acronyms are provided in the ‘Materials and Methods’ section. All measurements are in millimetres (mm).

**Holotype**. ZSIC 14304, adult male, from Subhong (24°58′34″N 92°47′45″E, 110 m asl), Cachar district, Assam state, India, collected by AD on 20 May 2008.

**Paratypes**. ZSIC 14305–14309, five adult males, and ZSIC 14310–14312, three adult females, collected along with the holotype.

**Referred specimens**. SDBDU 3918, an adult male and SDBDU 3919, an adult female, collected by AD along with the holotype; SDBDU 3572, an adult male, from Belonia, South district, Tripura, collected by AD and SG; SDBDU 3828, sub-adult male, from Chandel, Chandel district, Manipur, collected by SDB and SG.

**Etymology**. The species epithet, *aishani*, is an invariable feminine noun derived from the Sanskrit word ‘*aishani*’ or aiśānī (meaning north-east), referring to the Northeast regions of India where this frog was discovered.

**Diagnosis**. The new species is assigned to the genus *Micryletta* due to the following combination of morphological traits: small body size (SVL 22–28 mm); absence of vomerine teeth; prominent subarticular tubercles on fingers and toes; finger and toe tips slightly expanded in to small discs; and absence of webbing between fingers and toes (Dubois, 1987; Fei et al., 2009). *Micryletta aishani* sp. nov. differs from the other recognised species of the genus by the following suite of morphological characters: relatively small adult size (SVL 22.1–23.5 mm, male, *N* = 7; SVL 25.6–27.3 mm, female, *N* = 4); slender body; snout nearly truncate in dorsal and ventral view, acute in lateral view; tibiotarsal articulation reaching up to the level of armpit when stretched forward along the body axis; dorsal skin shagreened with minute spinules; outer metatarsal tubercles absent; webbing between toes absent; dorsum brown to reddish-brown with a faint brown median band extending from margins of the upper eye lids and tapering up to the vent, and few scattered blackish-brown spots on posterior parts of the back and near the groin; lateral surfaces of head blackish-brown; prominent blackish-brown streak extending from tip of the snout up to lower abdomen; ash-grey mottling along the margins of the upper and lower lip, extending up to the flanks and the limb margins; anterior and posterior parts of thigh, tarsus, and dorsal surfaces of hand and foot brown with ash-grey mottling; iris bicoloured, upper half light brown and lower half dark brown; belly ash-grey with a purplish tinge and brown mottling towards the margins.

**Description of holotype (measurements in mm)**. Adult male (SVL 22.8); head wider (HW 6.2) than long (HL 5.7); snout nearly truncate in dorsal and ventral view, acute in lateral view, its length (SL 2.7) longer than horizontal diameter of the eye (EL 2.2); loreal region vertical with rounded canthus rostralis; interorbital space flat, wider (IUE 2.7) than the upper eyelid (UEW 1.3) and internarial distance (IN 1.6); nostril closer to tip of the snout (NS 0.9) than the eye (EN 1.2); tympanum weakly-developed (TYD 0.4), less than one-fifth (18.2%) of the eye diameter (EL 2.2); supratympanic fold extending from posterior corner of eye to the shoulder weakly-developed; vomerine teeth absent; tongue small, oval, without papillae. Forearm (FAL 5.3) shorter than hand length (HAL 5.9); fingers without dermal fringes, finger length formula: I < II < IV < III, finger discs slightly wide compared to finger width (FD_I_ 0.4, FW_I_ 0.3; FD_II_ 0.4, FW_II_ 0.3; FD_III_ 0.5, FW_III_ 0.4; FD_IV_ 0.5, FW_IV_ 0.4), finger discs without grooves; subarticular tubercles prominent, oval, all present, subarticular tubercle formula: F_I_ 1, F_II_ 1, F_III_ 2, F_IV_ 2; single rounded supernumerary palmar tubercle at the base of fingers II, III, and IV; three metacarpal (palmar) tubercles; inner metacarpal tubercle distinct, rounded, small; outer metacarpal tubercle rounded, larger; medial metacarpal tubercle rounded, large; nuptial pads absent. Hind limbs slender, tibiotarsal articulation reaches up to the level of armpit (well below the eye) when stretched forward along the body axis; thigh length (TL 10.4) shorter than shank (SHL 10.7) and foot (FOL 11.4); distance from heel to tip of toe IV (TFOL 16.8); relative toe lengths: I < V < II < III < IV, toe discs slightly wide compared to toe width (TD_I_ 0.5, TW_I_ 0.4; TD_II_ 0.6, TW_II_ 0.4; TD_III_ 0.7, TW_III_ 0.5; TD_IV_ 0.6, TW_IV_ 0.4; TD_V_ 0.6, TW_V_ 0.4), toe discs without grooves; toes free of webbing; subarticular tubercles on toes prominent, oval, alternating with additional smaller tubercles, formula: T_I_ 1(L), T_II_ 1(S) + 1(L), T_III_ 1(S) + 1(L) + 1(S) + 1(L), T_IV_ 1(S) + 1(L) + 1(L) + 1(S) + 1(L), T_V_ (1S) + 1(L) + 1(S) + 1(L); single inner metatarsal tubercle (IMTL 0.6), oval-shaped, prominent, much shorter than half the length of toe I; outer metatarsal, and supernumerary metatarsal tubercles absent (Fig. 3).

Skin of snout, upper eyelids, sides of head, anterior and posterior parts of back, and upper and lower parts of flank shagreened with minute spinules; dorsal surfaces of forelimbs smooth, dorsal surfaces of hind limbs sparsely granular; ventral surface of throat smooth with minute spinules; chest, belly, and ventral surface of limbs smooth (Fig. 3).

**Colour of holotype**. *In life*. Dorsum reddish-brown with few scattered blackish-brown spots on posterior parts of the back and near the groin; a faint brown median band extending from margins of the upper eye lids and tapering up to the vent; lateral surfaces of head blackish-brown; a prominent blackish-brown streak extending from tip of the snout up to lower abdomen; ash-grey mottling along the margins of the upper and lower lip, extending up to the flanks, limb margins and dorsal surfaces of hand and foot; dorsal surface of forearms light reddish-brown; fingers brown with ash-grey mottling; dorsal surface of hind limbs reddish-brown with faint dark brown mottling, without dark transverse bands; toes I–IV brown with ash-grey mottling; groin, anterior and posterior parts of thigh, and lateral surfaces of shank and tarsus brown with ash-grey mottling; iris bicoloured, upper half light brown and lower half dark brown; ventral surface of throat greyish-brown; chest ash-grey with a purplish tinge and brown mottling; belly and fore- and hind limbs ash-grey with a purplish tinge and dark brown mottling towards the margins (Fig. 4). *In preservation*. Dorsum light greyish-brown with few scattered dark brown spots on posterior parts of the back, near the groin, and fore- and hind limbs; a faint greyish-brown median band extending from margins of the upper eye lids and tapering up to the vent; a dark brown streak extending from tip of the snout up to lower abdomen; lateral abdominal surfaces light grey with brown mottling; ventral surface of throat dark greyish-brown; chest light greyish-brown with brown mottling; belly, hand and foot greyish-brown with dark brown mottling towards the margins (Fig. 3).

**Variations**. Morphometric data from seven adult males and four adult females, including the holotype, is given in Table 3. Overall, the colour and meristic characters of the paratypes are similar to the holotype. Skin texture varies based on the sex: males with more prominent spinules on dorsal skin and throat. Ventral colouration varies from ash-grey to light purple with less or more prominent mottling (Figs. 4 and 5).

**Secondary sexual characters**. Male: single gular pouch; female (ZSIC 14310): pigmented eggs (diameter 1.1 ± 0.4 mm, *N* = 10).

**Distribution and natural history**. *Micryletta aishani* sp. nov. is currently known from three Northeast Indian states of Assam, Tripura, and Manipur (Fig. 1). At the type locality (Subhong), we came across a large aggregation of calling males in the month of May at around 8.30 pm. Individuals were calling from waterlogged fern banks located at the base of a small valley between hillocks (locally called *Tillas*). The area is characterised by degraded forest with areca nut plants and beetle vine cultivation located close to a human settlement (∼1 km). Two females were collected from the exposed slopes of the tillas close to the congregation. The species seems to have a narrow breeding season with very specific requirements as we failed to record any individuals either before waterlogging (during early April) or once the fern banks began to submerge in water (by June). Among other anurans in the same habitat, we recorded *Microhyla mymensinghensis* Hasan, Islam, Kuramoto, Kurabayashi, and Sumida, *Kurixalus* sp., *Rhacophorus smaragdinus* Blyth, *Raorchestes* sp., and *Humerana humeralis* Boulenger. At Tripura and Manipur, individuals were collected from ground or leaf litter near shallow streams and marshy areas covered with thick vegetation. Collection sites were located in degraded secondary forest areas close to human settlement.

**Morphological comparison**. *Micryletta aishani* sp. nov. can be distinguished from other congeners by the outline of its snout nearly truncate in dorsal and ventral view, except in *M. steinegeri*; and prominent ash-grey colouration on the flanks, groin, anterior and posterior parts of thigh, lateral surfaces of shank and tarsus, toes I–IV, limb margins, and ventral surfaces.

Specifically, *M. aishani* sp. nov. differs from *M. erythropoda* by its relatively smaller adult snout-vent size, SVL 22.1–23.5 mm in males, *N* = 7, SVL 25.6–27.3 mm in females, *N* = 4 (vs. SVL up to 30 mm); outline of the snout nearly truncate in dorsal and ventral view (vs. obtuse); dorsum with few scattered blackish-brown spots on posterior part of the back and near the groin (vs. dark contrasting round or irregular shape spots irregularly scattered throughout the dorsum); dorsal skin shagreened with minute spinules (vs. skin smooth, slightly granulated on the lateral sides); tympanum much smaller than the eye diameter, TYD/EL 18.2–28.6% in males, TYD 32–41% in females (vs. relatively larger, equal to the half length of eye); tibiotarsal articulation reaching up to the level of armpit when stretched forward along the body axis (vs. tibiotarsal joint not reaching the hind part of tympanum); absence of outer metatarsal tubercles (vs. present); and webbing absent between toes (vs. rudimentary).

*Micryletta aishani* sp. nov. differs from *M. inornata* by its larger adult snout-vent size, SVL 22.1–23.5 mm in males, *N* = 7, SVL 25.6–27.3 mm in females, *N* = 4 (vs. smaller, SVL 16.8–20.5 mm in males, *N* = 3, SVL 19.5 mm in female, *N* = 1); outline of the snout nearly truncate in dorsal and ventral view (vs. nearly rounded); snout longer than the eye diameter, SL/EL 1.22–1.33 in males, *N* = 7, SL/EL 1.08–1.36 in females, *N* = 4 (vs. shorter, SL/EL 0.86–0.91 in males, *N* = 3, SL/EL 0.92 in female, *N* = 1); tibiotarsal articulation reaching up to the level of armpit when stretched forward along the body axis (vs. up to the level of eye); dorsal colouration reddish-brown with few scattered blackish-brown spots on posterior parts of the back and near the groin (vs. dark brown or brownish-grey with a silver tinge, and irregular blackish-brown blotches); dorsal skin shagreened with minute spinules (vs. smooth, covered with small tubercles or warts); anterior and posterior parts of thigh, lateral surfaces of shank and tarsus, and toes I–IV brown with ash-grey mottling (vs. light flesh-red without mottling); and ventral surface of belly, fore- and hind limbs ash-grey with a purplish tinge and brown mottling towards the margins (vs. light reddish-grey without mottling).

*Micryletta aishani* sp. nov. differs from *M. nigromaculata* by the outline of its snout nearly truncate in dorsal and ventral view (vs. rounded); eyes shorter than snout, EL/SL 0.75–0.82 in males, *N* = 7, EL/SL 0.73–0.93 in females, *N* = 4 (vs. relatively longer, EL/SL 0.86–1.07 in males, *N* = 18, EL/SL 0.91–1.03 in females, *N* = 3); males with a relatively smaller tympanum than the eye, TYD/EL 0.14–0.29 (vs. relatively larger, TYD/EL 0.36–0.46, *N* = 18); supratympanic fold weakly-developed (vs. thick and glandular); tibiotarsal articulation reaching up to the level of armpit when stretched forward along the body axis (vs. reaching up to the level of eye); dorsal skin shagreened with minute spinules (vs. slightly granular with small round flattened tubercles); dorsum reddish-brown with a faint median band (vs. dark brown irregular hourglass-shaped pattern having orange edges); a prominent dark blackish-brown streak from tip of the snout up to the lower abdomen on either side (vs. scattered dark spots or patches); ventral surface of belly ash-grey with a purplish tinge and brown mottling towards the margins (vs. whitish with indistinct light grey marbling).

*Micryletta aishani* sp. nov. differs from *M. steinegeri* by its relatively larger snout, SL/HL 0.43–0.52 in males, *N* = 7, SL/HL 0.39–0.47 in females, *N* = 4 (vs. shorter, SL/HL 0.34–0.38 in males, *N* = 3, SL/HL 0.37–0.38 in females, *N* = 2); eyes smaller than the snout, EL/SL 0.75–0.82 in males, *N* = 7, EL/SL 0.73–0.93 in females, *N* = 4 (vs. larger, EL/SL 1.04 in males, *N* = 3, EL/SL 1.07–1.14 in females, *N* = 2); tibiotarsal articulation reaching up to the level of armpit when stretched forward along the body axis (vs. reaching up to the level of tympanum); dorsum reddish-brown with few scattered blackish-brown spots on posterior parts of the back and near the groin (vs. dark grey to violet with irregular dark blotches or speckles); dorsal skin shagreened with minute spinules (vs. smooth to shagreened); anterior and posterior parts of thigh, lateral surfaces of shank and tarsus, and toes I–IV brown with ash-grey mottling (vs. light flesh-red without mottling); ventral surface of belly, fore- and hind limbs ash-grey with a purplish tinge and brown mottling towards the margins (vs. greyish-white); and webbing absent between toes (vs. rudimentary).

Furthermore, the new species *M. aishani* sp. nov. cannot be confused with another available nomen, *M. inornata lineata*, currently under the synonymy of *M. inornata*, due to its reddish-brown dorsum with a faint median band (vs. greyish-brown with three straight continuous or broken lines); snout longer than the horizontal diameter of eye, SL/EL 1.22–1.33 in males, *N* = 7, SL/EL 1.08–1.36 in females, *N* = 4 (vs. snout distinctly shorter than the eye length); dorsal skin shagreened with minute spinules (vs. smooth); and tibiotarsal articulation reaching up to the level of armpit when stretched forward along the body axis (vs. reaching up to the eye).

### Redescription and lectotype designation

**Figure 6:**
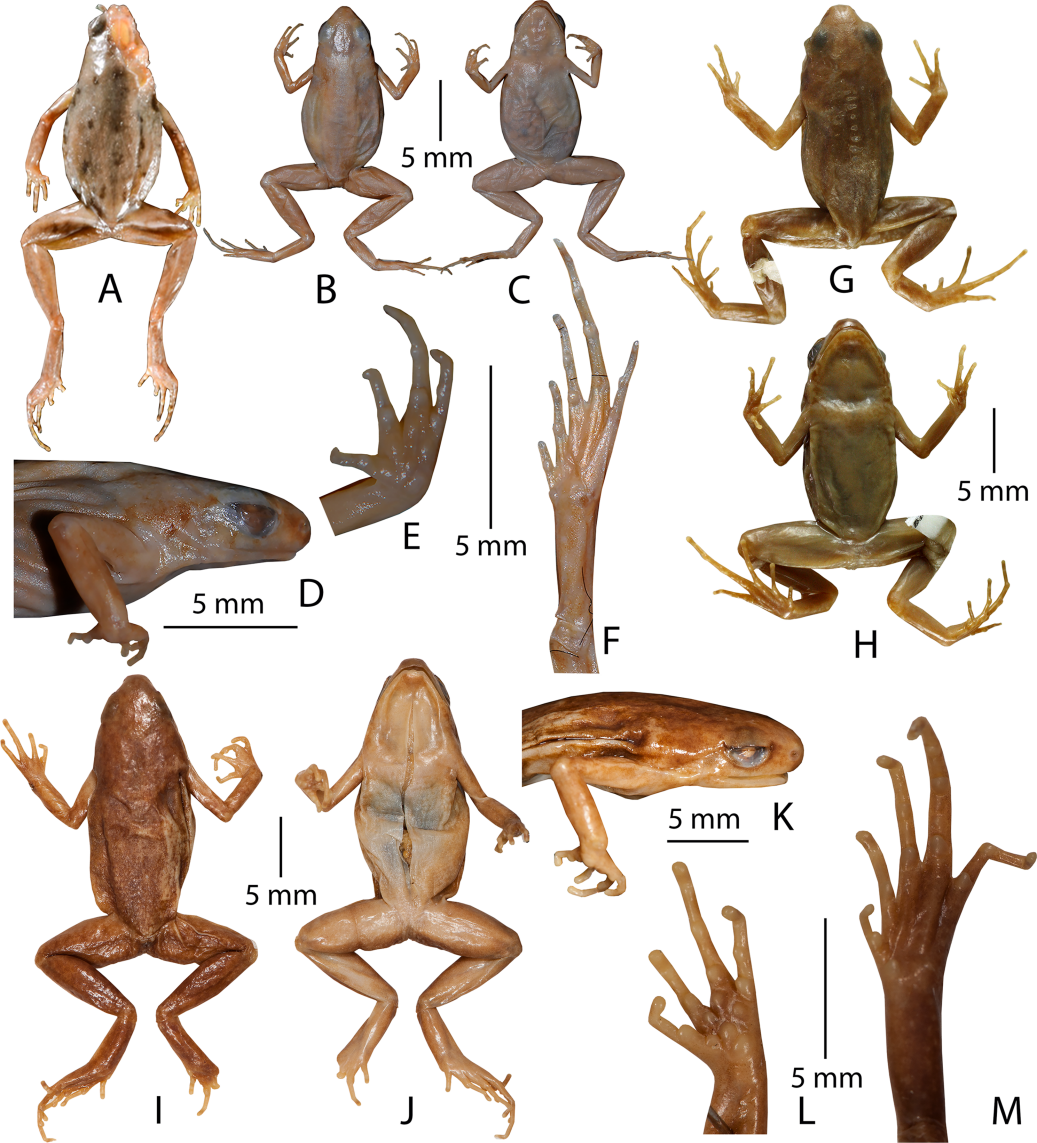
Type specimens of *Micryletta* species in preservation. (A) Holotype (NA 3276) of *Microhyla erythropoda* (= *Micryletta erythropoda*), dorsal view. (B–F) Lectotype (NHM 1889.11.12.4 (ex. BNHS 1947.2.11.74)) of *Microhyla inornata* (= *Micryletta inornata*). (B) Dorsal view. (C) Ventral view. (D) Lateral view of head. (E) Ventral view of hand. (F) Ventral view of foot. (G–H) Holotype (FMNH 178245) of *Microhyla inornata lineata* (= *Micryletta inornata lineata*). (G) Dorsal view. (H) Ventral view. (I–M) Lectotype (NHM 1909.10.29.92 (ex. BMNH 1947.2.11.76)) of *Microhyla steinegeri* (= *Micryletta steinegeri*). (I) Dorsal view. (J) Ventral view. (K) Lateral view of head. (L) Ventral view of hand. (M) Ventral view of foot.

***Micryletta inornata* (Boulenger, 1890)**
Deli Paddy Frog
(Fig. 6; Table 3)

**Lectotype**. By present designation, NHM 1889.11.12.4 (ex. BMNH 1947.2.11.74), an adult female, from “Deli, Sumatra”, Indonesia.

**Paralectotype**. NHM 1889.11.12.30 (ex. BMNH 1947.2.11.75), an adult male, from “Deli, Sumatra”, Indonesia.

**Diagnosis**. *Micryletta inornata* differs from all other members of the genus by the following suite of morphological characters: smaller adult size, SVL 16.8–20.5 mm in males, *N* = 3, SVL 19.5 mm in female, *N* = 1; outline of the snout nearly rounded in dorsal and ventral view; tibiotarsal articulation reaching up to the level of eye when stretched forward along the body axis; dorsum brownish-grey with a silver tinge and irregular blackish-brown blotches; lateral surfaces of head blackish-brown with a silver white line along the upper lip; ventral surface of belly light reddish-grey without mottling; and its distribution restricted to the islands of Sumatra, Indonesia (Alhadi et al., 2019). For comparison with other congeners, see the ‘Morphological comparison’ section of *M. aishani* sp. nov.

**Figure 7:**
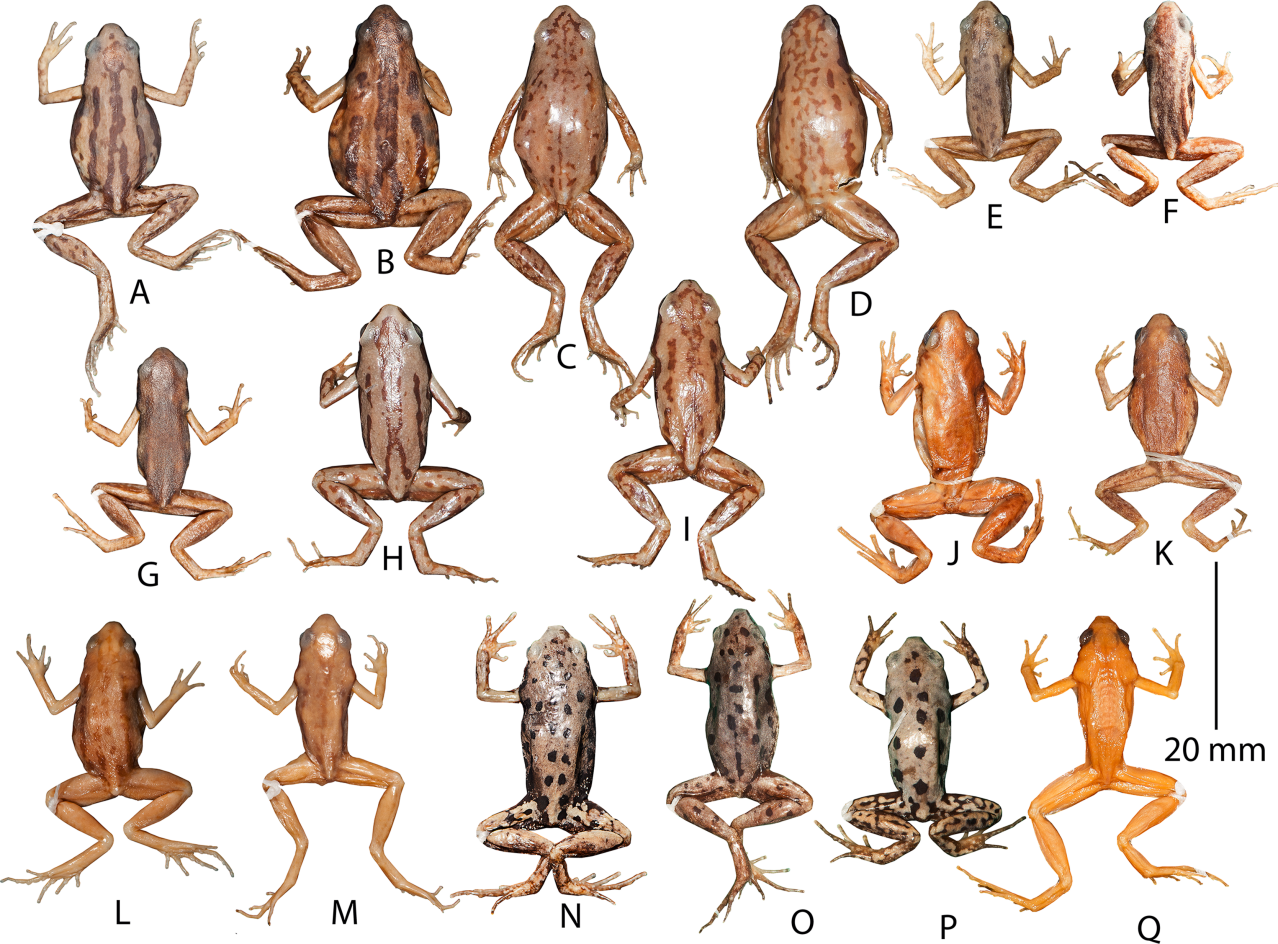
Dorsal markings in preserved museum specimens identified as *Micryletta* ‘*inornata*’ from regions across Southeast Asia. (A) MNHNP 1987.2472 (SVL 25.2 mm, female), Thaicaude, Khao Choug, Thailand. (B) MNHNP 1987.2619 (SVL 24.4 mm, female), Thaicaude, Khao Choug, Thailand. (C) NHM 1897.10.8.69–77 (no individual number, female), Siam (= Thailand). (D) NHM 1897.10.8.69–77 (no individual number, female), Siam (= Thailand). (E) MNHNP 1997.1441 (SVL 18.4 mm, male), Chiang Mai, Thailand. (F) MNHNP 1987.2477 (SVL 18.9 mm, male), Thaicaude, Khao Choug, Thailand. (G) MNHNP 1987.2542 (SVL 17.9 mm, male), Thaicaude, Khao Choug, Thailand. (H) NHM 1987.10.69–77 (no individual number, male), Siam (= Thailand). (I) NHM 1987.10.69–77 (no individual number, male), Siam (= Thailand). (J) NHM 1974.3285 (SVL 24.4 mm, female), Kuala Tahan, Panag, Malaysia. (K) NHM 1974.3306 (SVL 19.5 mm, male), Kuala Tahan, Panag, Malaysia. (L) MNHNP 1997.4109 (SVL 28.1 mm, female), Nam Khan, Province de Bokeo, Laos. (M) MNHNP 2005.0179 (SVL 19.3 mm, male), Long Mai Kao, Province de Phongsaly, Laos. (N) MNHNP 2010.0765 (SVL 23.7 mm, male), Sraaem, Preah Vihear, Cambodia. (O) MNHNP 2010.0767 (SVL 25.4 mm, female), Sraaem, Preah Vihear, Cambodia. (P) MNHNP 2010.0769 (SVL 21.2 mm, male), Sraaem, Preah Vihear, Cambodia. (Q) NHM 1986.670 (SVL 20.0 mm, male), Kenting park, Ping-tung countty, Taiwan.

**Rationale for lectotypification**. This species was originally described (Boulenger, 1890) based on three specimens (two males and one female) from “Deli, Sumatra”. Subsequently, it was reported to occur widely across Mainland Southeast Asia (Thailand, Malaysia, Vietnam, Laos, Cambodia, and Myanmar) with few isolated records from South Asia (Manipur and Andaman Islands, India) and East Asia (southern China and Taiwan Island) (e.g. Berry, 1975; Pillai, 1977; Ohler, Swan & Daltry, 2002; Nguyen, Ho & Nguyen, 2005; Stuart & Emmett, 2006; Guo, Yang & Li, 2009; Mathew & Sen, 2010; Fei, Ye & Jiang, 2010; Frost, 2018; Poyarkov et al., 2018). However, the absence of any new collections of this species from its type locality or close vicinities since its original description had propagated confusions surrounding the identity of this taxon, as well as deterred meaningful taxonomic studies in the genus (e.g. Matsui et al., 2011; Poyarkov et al., 2018), until its recent rediscovery (Alhadi et al., 2019). Although, the distribution of this taxon has currently been restricted to Sumatra (Alhadi et al., 2019), the taxonomic status of a large number of populations identified as *Micryletta inornata* from other regions for over a century remains to be validated. Most reports of larger-sized (>20 mm) male as well as female specimens identified as *M. inornata* from other regions (Fig. 7) are all likely to be misidentifications requiring confirmation based on detailed morphological and molecular studies. Hence, in order to avoid any confusions surrounding the identity of *Micryletta inornata (sensu stricto)* and to define it objectively, in accordance with Article 74 of International Code of Zoological Nomenclature (International Commission on Zoological Nomenclature, 1999) we find it important to designate a lectotype from the syntypes (one of which remains unavailable) so that it can become the unique name bearer of this taxon.

We examined the two currently available specimens (one male, NHM 1889.11.12.30 (ex. BMNH 1947.2.11.75) and one female, NHM 1889.11.12.4 (ex. BMNH 1947.2.11.74)) at NHM, London. The female syntype NHM 1889.11.12.4 (ex. BMNH 1947.2.11.74) from “Deli, Sumatra” agrees with the original description, with respect to snout-vent size (“From snout to vent 20 million”) and most other characters. Since this specimen was found to be in a relatively good condition, apart from bleached dorsal skin, we herein designate NHM 1889.11.12.4 (ex. BMNH 1947.2.11.74), an adult female, as the lectotype of *Microhyla inornata* Boulenger, 1890 (= *Micryletta inornata*), in order to stabilise the nomenclatural status of this species. The lectotype description provided below is largely consistent with the original description.

**Description of lectotype (measurements in mm)**. Adult female (SVL 19.5); head wider (HW 5.9) than long (HL 5.3); snout nearly rounded in dorsal and ventral view, acute in lateral view, its length (SL 2.2) slightly shorter than horizontal diameter of the eye (EL 2.4); loreal region acute with rounded canthus rostralis; interorbital space flat, wider (IUE 2.5) than the upper eyelid (UEW 1.3) and internarial distance (IN 1.5); tympanum weakly-developed, supratympanic fold extending from posterior corner of eye to the shoulder weakly-developed; vomerine teeth absent; tongue small, oval, without papillae. Forearm (FAL 4.8) nearly equal to hand length (HAL 4.7); fingers without dermal fringes, finger length formula: I < II < IV < III, finger discs slightly wide compared to finger width, discs without grooves; fingers free of webbing; subarticular tubercles prominent, oval, all present, subarticular tubercle formula: F_I_ 1, F_II_ 1, F_III_ 2, F_IV_ 2; single rounded supernumerary palmar tubercles at the base of fingers II, III, and IV; three metacarpal (palmar) tubercles; inner metacarpal tubercle distinct, rounded, smaller; outer metacarpal tubercle rounded, larger; medial metacarpal tubercle rounded, large; nuptial pad absent. Hind limbs slender; tibiotarsal articulation reaches up to the level of eye when stretched forward along the body axis; thigh length (TL 8.1) shorter than shank (SHL 8.6) and foot (FOL 8.6); toe discs slightly wide compared to toe width, discs without grooves; toes free of webbing; subarticular tubercles on toes prominent, oval; single inner metatarsal tubercle (IMTL 0.4), prominent, rounded; outer metatarsal tubercle, and supernumerary tubercles absent.

Skin of dorsum smooth to shagreened, covered with small scattered tubercles; ventral surfaces smooth. Most of the skin and colour characters cannot be reliably determined due to the bleached skin of the specimen.

***Micryletta steinegeri* (Boulenger, 1909)**
Stejneger’s Paddy Frog
(Fig. 6; Table 3)

**Lectotype**. By present designation, NHM 1909.10.29.92 (ex. BMNH 1947.2.11.76), an adult female, from “Kanshirei”, “Formosa” (= Taiwan).

**Paralectotypes**. NHM 1909.10.29.94 (ex. BMNH 1947.2.11.78), NHM 1909.10.29.95 (ex. BMNH 1947.2.11.79), NHM 1909.10.29.96 (ex. BMNH 1947.2.11.80), three adult males, and NHM 1909.10.29.93 (ex. BMNH 1947.2.11.77), an adult female, from “Kanshirei”, “Formosa” (= Taiwan).

**Diagnosis**. *Micryletta steinegeri* differs from all other members of the genus by the following suite of morphological characters: SVL 22.6–23.5 mm, *N* = 3 in males, 27.0–30.1 mm, *N* = 2 in females; outline of snout rounded to truncate in dorsal and ventral view; tibiotarsal articulation reaching up to the level of tympanum when stretched forward along the body axis; dorsum dark grey to violet with irregular dark blotches or speckles; dorsal skin smooth to shagreened; anterior and posterior parts of thigh light flesh-red without mottling; belly, fore- and hind limbs greyish-white; and rudimentary webbing between toes. For comparison with other congeners, see the ‘Morphological comparison’ section of *M. aishani* sp. nov.

**Rationale for lectotypification**. The original description was based on five specimens from “Kanshirei”, “Formosa” (= Taiwan) (Boulenger, 1909). We examined all the five specimens (three males, NHM 1909.10.29.94 (ex. BMNH 1947.2.11.78), NHM 1909.10.29.95 (ex. BMNH 1947.2.11.79), NHM 1909.10.29.96 (ex. BMNH 1947.2.11.80), and two females, NHM 1909.10.29.92 (ex. BMNH 1947.2.11.76), NHM 1909.10.29.93 (ex. BMNH 1947.2.11.77)) at NHM, London. The female syntype NHM 1909.10.29.92 (ex. BMNH 1947.2.11.76) from “Kanshirei”, exactly agrees with the original description in terms to snout-vent size (“Total length 30 mm”) and most other characters. Since this specimen is likely to have been the one used by Boulenger (1909) to describe the species, in accordance with Article 74 of International Code of Zoological Nomenclature (International Commission on Zoological Nomenclature, 1999) it is advisable for it to become the unique name bearer of the taxon and the standard for its application. This specimen was also found to be in a relatively good condition. Therefore, we herein designate NHM 1909.10.29.92 (ex. BMNH 1947.2.11.76), an adult female, as the lectotype of *Microhyla steinegeri* Boulenger, 1909 (= *Micryletta steinegeri*), in order to stabilise the nomenclatural status of this species. The lectotype description provided below is largely consistent with the original description.

**Description of lectotype (measurements in mm)**. Adult female (SVL 30.1); head wider (HW 8.4) than long (HL 7.8); snout rounded to truncate in dorsal and ventral view, acute in lateral view, its length (SL 2.9) shorter than horizontal diameter of the eye (EL 3.3); loreal region acute with rounded canthus rostralis; interorbital space flat, wider (IUE 3.8) than the upper eyelid (UEW 1.8) and internarial distance (IN 2.1); tympanum weakly-developed, supratympanic fold extending from posterior corner of eye to the shoulder weakly-developed; vomerine teeth absent; tongue small, oval, without papillae. Forearm (FAL 6.5) slightly shorter than hand length (HAL 6.7); fingers without dermal fringes, finger length formula: I < IV < II < III, finger discs slightly wide compared to finger width, discs without grooves; subarticular tubercles prominent, oval, all present, subarticular tubercle formula: F_I_ 1, F_II_ 1, F_III_ 2, F_IV_ 2; single rounded supernumerary palmar tubercles at the base of fingers II, III, and IV; three metacarpal (palmar) tubercles; inner metacarpal tubercle distinct, rounded, smaller; outer metacarpal tubercle rounded, larger; medial metacarpal tubercle rounded, large; nuptial pad absent. Hind limbs slender, tibiotarsal articulation reaches up to the level of tympanum when stretched forward along the body axis; thigh length (TL 12.1) shorter to shank (SHL 13.0) and foot (FOL 13.6); toe discs slightly wide compared to toe width, discs without grooves; toes with rudimentary webbing; subarticular tubercles on toes prominent, oval; single inner metatarsal tubercle (IMTL 0.6), prominent, rounded; outer metatarsal tubercle and supernumerary tubercles absent.

Skin of dorsum smooth to shagreened; ventral surfaces smooth.

## Discussion

The discovery of a new species of *Micryletta* from Northeast India highlights that although this region is considered as a ‘gateway’ between the Indian subcontinent and Eurasia, it harbours some unique microendemics, which are often overlooked due to presumed similarities with faunal elements in adjoining Southeast and East Asia (Mani, 1995; Kamei et al., 2012). It is therefore important to extensively document the biodiversity of Northeast Indian regions to gather a proper understanding of extant life forms and the evolutionary relationships between South and Southeast Asian faunal elements.

Although rare to find due to its extremely short breeding season, the new *Micryletta* species is likely to be more widely distributed and common within the Northeast states. Apart from the confirmed records of *M. aishani* sp. nov. from regions south of River Brahmaputra in Assam, Manipur, and Tripura, one of us (AD) has also observed an unidentified *Micryletta* population (not collected) north of the river in Manas National Park, Assam. Further studies are required to ascertain the taxonomic status of this population as well as the role of physical barriers such as River Brahmaputra that divides Northeast India into two distinct biogeographical regions (Mani, 1995). After this study, it is now certain that the geographical distribution of genus *Micryletta* covers most of Indo-Burma biodiversity hotspot (including Northeast India) and extends towards Sundaland; the former appearing to be the major centre for species-level diversification. Further surveys in adjoining Himalayan regions will be necessary to understand the distribution limits of *Micryletta*. The biogeography of the genus could also shed light on various barriers of dispersal for amphibian species among the three adjoining, yet unique, biodiversity hotspots.

The present study also provides taxonomic insights on this morphologically complex genus of microhylid frogs with relatively conserved morphology at species-level despite considerable genetic differentiation (Table 2). As evident from the available molecular data (e.g. Vences, 2000; Ziegler, 2000; Matsui et al., 2011; Kurabayashi et al., 2011; De Sá et al., 2012; Blackburn et al., 2013; Grosjean et al., 2015; Peloso et al., 2016; Poyarkov et al., 2018; Alhadi et al., 2019), the currently recognised diversity in the genus *Micryletta* is underestimated. Several candidate species (*Micryletta* sp. A, *Micryletta* sp. B, *M*. cf. *steinegeri*, and *M*. cf. *nigromaculata*) exist among populations identified as *M*. ‘*inornata*’ from Southeast Asian regions of Thailand, Laos, and Vietnam (Fig. 2). Another two populations from southern Thailand and Myanmar, one of which was previously identified as the subspecies ‘*M. inornata lineata*’ (Matsui et al., 2011), were found more closely related to *M. erythropoda* with divergence of 2.5%, than the typical *M. inornata* or any other clade referred to as *M*. cf. *inornata* (Alhadi et al., 2019). Hence, the clade *M*. cf. *inornata lineata* is likely to represent a valid species, however, a resolution of its identity, as well as the taxonomic status of *M. erythropoda*, remains pending until availability of topotypic material of *M. inornata lineata* (Poyarkov et al., 2018; Alhadi et al., 2019).

Until recently, the phylogenetic position of the type species, *Micryletta inornata*, was unknown due to the absence of genetic data for populations from the type locality in Sumatra (Alhadi et al., 2019). Eventhough our study confirms that populations previously identified as *M*. ‘*inornata*’ from Northeast India represent another species (*M. aishani* sp. nov.), the status of other widely reported *M*. cf. ‘*inornata*’ populations from mainland Southeast Asia (e.g. Thailand, Vietnam, and Laos) still remains uncertain. However, the available data clearly shows *M. inornata*, as presently understood in a phylogenetic framework, to be a complex of multiple species (e.g. Matsui et al., 2011; Poyarkov et al., 2018; Alhadi et al., 2019), either requiring assignment to other available names (such as *M. steinegeri*, *M. inornata lineata*, and *M. nigromaculata*) or new names (for *Micryletta* sp. A, and *Micryletta* sp. B, *M*. cf. *steinegeri*, and *M*. cf. *nigromaculata*). Specifically, *M. steinegeri* that is presently considered endemic to the island of Taiwan shows considerably lower genetic divergence from mainland Vietnamese populations referred to as *M*. cf. *steinegeri* (“*M*. cf. *inornata* B” in Poyarkov et al., 2018; “*M*. cf. *inornata*” in Alhadi et al., 2019). Therefore, the latter is more likely to represent new records of *M. steinegeri* from Mainland Southeast Asia.

Due to the prevailing taxonomic uncertainities, proper morphological identification of all known *Micryletta* populations, complemented with the molecular knowledge, is necessary for further meaningful studies on this group. In the present study, we examined several museum specimens identified as *M*. ‘*inornata*’ from countries across Southeast Asia (Fig. 7) and found them to differ in their overall external morphology. Currently, *M. inornata* is the smallest sized member of the genus and known only from Sumatra. However, many of the examined museum specimens of *M*. ‘*inornata*’ show considerable morphological differences from the types (NHM 1889.11.12.30 (ex. BMNH 1947.2.11.75) and NHM 1889.11.12.4 (ex. BMNH 1947.2.11.74)) as well as the characters discussed in the original description (Boulenger, 1890), and appear to be misidentifications, possibly referring to *M. erythropoda* (e.g. MNHNP 2010.0765, MNHNP 2010.0767, MNHNP 2010.0769, from Cambodia) and *M. inornata lineata* (e.g. MNHNP 1987.2472, MNHNP 1987.2619, NHM 1987.10.69–77 with no individual numbers from ‘Siam’) (Fig. 7). As noted by previous researchers, members of the genus *Micryletta* have contrasting dorsal markings (e.g. Wang, Wu & Yu, 1989; Matsui et al., 2011; Poyarkov et al., 2018). However, Wang, Wu & Yu (1989) reported various colour morphs of *M*. ‘*inornata*’ categorised in to four types of dorsal markings, suggesting that dorsal colour and markings may have little taxonomic value in identification of *Micryletta* species. At the same time, they discussed size variations with Taiwanese populations shown to be larger than Thailand populations (Wang, Wu & Yu, 1989). Hence, the confusing morphology combined with overlapping distribution ranges of species makes delineation of the yet unidentified specimens and/or phylogenetically distinct lineages difficult. The rediscovery of *M. inornata* from Sumatra (Alhadi et al., 2019) and the proper identification of Northeast Indian populations in the present study, will aid future studies in providing a further resolution to the complicated taxonomy of *Micryletta*, consequently also enabling a better understanding of the patterns of diversification and geographical distribution in this group. A wider taxon sampling throughout the range of the genus in South, Southeast, and East Asia, especially from unexplored intervening regions, will also be necessary to gather a better understanding of intra- and interspecific variations, both morphologically and genetically (e.g. Matsui et al., 2011; Poyarkov et al., 2018). This could further result in the discovery of unidentified lineages and fill in the gaps to fully reconstruct the evolutionary history of this enigmatic group of microhylid frogs.

## Conclusions

Our description of a new species of *Micryletta* from Northeast India contributes to a better understanding of the diversity in this genus. The discovery also genetically validates the presence of the genus *Micryletta* in India and the westward extension of its geographical range within South Asia. The study provides evidence for genotypic and phenotypic distinctness of the new species from all the previously recognised congeners, designates lectoptypes for nomenclatural stability of two previously known species, and confirms the presence of additional undescribed lineages within the genus. Altogether, our work will facilitate future taxonomic, phylogenetic, and biogeographical studies in this microhylid group.

## Abbreviations

ZSIC: Zoological Survey of India, Kolkata, India
NHM: Natural History Museum, London, United Kingdom
BMNH: British Museum of Natural History, London, United Kingdom
FMNH: Field Museum of Natural History, Chicago, USA
MNHNP: Museum National d’Histoire Naturelle, Paris, France
ZMMU: Zoological Museum of Lomonosov Moscow State University, Moscow, Russia
DTU: Duy Tan University, Vietnam
HT: Holotype
PT: Paratype
LT: Lectotype
PLT: Paralectotype
ST: Syntype
TT: Topotype
RS: Referred specimen
AD: Abhijit Das
SG: Sonali Garg
SDB: S. D. Biju.

## Additional Information and Declarations

### Field Study Permissions

The following information was supplied relating to field study approvals (i.e. approving body and any reference numbers): Fieldwork and sampling were conducted with permissions from responsible authorities in the State Forest Departments of Assam, Tripura, and Manipur (Study permits: No. FWLG/455; No. F.8(163)/FOR-WL; No. 3/22/2016-WL).

### DNA Deposition

The following information was supplied regarding the deposition of DNA sequences: The DNA sequence is available at GenBank (MK889218).

### Data Availability

The following information was supplied regarding data availability: Type specimens are deposited at Zoological Survey of India, Kolkata (ZSIC) under accession numbers ZSIC 14304–14312. The newly generated DNA sequence is available at GenBank: MK889218.

### New Species Registration

The following information was supplied regarding the registration of a newly described species: Publication LSID: urn:lsid:zoobank.org:pub:F0C54D63-0B29-470F-BDCD-72F7E2511C51. *Micryletta aishani* sp. nov. LSID: urn:lsid:zoobank.org:act:A8DCDA95-6D6A-4710-9C4D-FC00881032EE.
